# Assessing causal relationships between diabetes mellitus and idiopathic pulmonary fibrosis: a Mendelian randomisation study

**DOI:** 10.1136/thorax-2024-221472

**Published:** 2024-11-29

**Authors:** Samuel T Moss, Cosetta Minelli, Olivia C Leavy, Richard J Allen, Nick Oliver, Louise V Wain, Gisli Jenkins, Iain Stewart

**Affiliations:** 1 National Heart and Lung Institute, Imperial College London, London, UK; 2 Department of Population Health Sciences, University of Leicester, Leicester, UK; 3 NIHR Leicester Biomedical Research Centre, Leicester, East Midlands, UK; 4 NIHR Leicester Biomedical Research Centre, University of Leicester, Leicester, UK; 5 Department of Metabolism, Digestion and Reproduction, Imperial College London, London, UK; 6 National Heart & Lung Institute, Imperial College London, London, UK

**Keywords:** Idiopathic pulmonary fibrosis, Clinical Epidemiology

## Abstract

**Background:**

Idiopathic pulmonary fibrosis (IPF) is a disease of progressive lung scarring. There is a known association between diabetes mellitus (DM) and IPF, but it is unclear whether a causal relationship exists between these traits.

**Objectives:**

The objectives of this study are to examine causal relationships among DM, diabetes-associated traits and IPF using a Mendelian randomisation approach.

**Methods:**

Two-sample MR approaches, including bidirectional inverse-variance weighted random effects and routine sensitivity models, used genetic variants identified from genome-wide association studies for type 1 diabetes (T1D), type 2 diabetes (T2D), glycated haemoglobin level (HbA1c), fasting insulin level and body mass index (BMI) to assess for causal effects of these traits on IPF. Further analyses using pleiotropy-robust and multivariable MR (MVMR) methods were additionally performed to account for trait complexity.

**Results:**

Results did not suggest that either T1D (OR=1.00, 95% CI 0.93 to 1.07, p=0.90) or T2D (1.02, 0.93 to 1.11, p=0.69) are in the causal pathway of IPF. No effects were suggested of HbA1c (1.19, 0.63 to 2.22, p=0.59) or fasting insulin level (0.60, 0.31 to 1.15, p=0.12) on IPF, but potential effects of BMI on IPF were indicated (1.44, 1.12 to 1.85, p=4.00×10^−3^). Results were consistent in MVMR, although no independent effects of T2D (0.91, 0.68 to 1.21, p=0.51) or BMI (1.01, 0.94 to 1.09, p=0.82) on IPF were observed when modelled together.

**Conclusions:**

This study suggests that DM and IPF are unlikely to be causally linked. This comorbid relationship may instead be driven by shared risk factors or treatment effects.

**WHAT IS ALREADY KNOWN ON THIS TOPIC**

- Idiopathic pulmonary fibrosis is associated with diabetes mellitus epidemiologically, but it is unclear if these traits are linked by causal effects.

**WHAT THIS STUDY ADDS**

- Idiopathic pulmonary fibrosis and diabetes mellitus are unlikely to be causally linked, suggesting that shared environmental risk factors or treatment effects may drive this comorbid relationship.

**HOW THIS STUDY MIGHT AFFECT RESEARCH, PRACTICE, OR POLICY**

- Further research investigating the relationship between diabetes mellitus and idiopathic pulmonary fibrosis should focus on potential shared risk factors and treatment effects such as corticosteroid use.

## Introduction

 Idiopathic pulmonary fibrosis (IPF) is a chronic disease characterised by progressive and uncontrolled scarring of the lung. The development of fibrosis in IPF reduces lung function over time and is typically fatal within 3–5 years of diagnosis. The mechanisms underlying IPF risk and progression are not fully understood.

Diabetes mellitus (DM) is a group of diseases characterised by hyperglycaemia due to disrupted insulin production, reduced effectiveness of secreted insulin, or a combination of both. Over 400 million people have DM globally and incidence has been shown to be increasing.^1^ Type 1 diabetes is caused by autoimmune destruction of pancreatic beta cells and subsequent insulin deficiency.^2^ Type 2 diabetes (T2D) is a long-term condition characterised by insulin resistance, dysregulation of blood glucose levels, and a progressive decline in beta cell function.^3^ Studies show increased prevalence of DM in patients with IPF when compared with controls^4^ and comorbid DM has been shown to be associated with increased mortality in patients with IPF.^5^ As current studies assessing this association have primarily used mixed DM cohorts, it is unclear if prevalence of DM in patients with IPF varies with DM subtype.

The potential mechanisms by which DM may be causally associated with IPF are complex. First, immune dysregulation and infection are important factors in IPF development.^6^ Hyperglycaemia has been linked to immune dysfunction, chronic low-grade inflammation, increased infection rate and worse immune-related clinical outcomes in critically ill patients.^7^ It is also plausible that hyperglycaemia may contribute to IPF risk through key fibrotic pathways including disrupted DNA repair mechanisms, increased cellular senescence caused by increased oxidative stress and activation of transforming growth factor-β (TGF-β) signalling.^8 9^ The potential relationship between T2D and IPF is further complicated by metabolic factors becoming dysregulated both due to T2D and during T2D disease development. Factors such as chronic hyperglycaemia, obesity and dysregulated insulin signalling are involved in the development of T2D^10^ and may independently drive IPF pathogenesis.

Mendelian randomisation (MR) is a methodology that aims to determine whether two associated variables have a causal relationship. This is achieved by using genetic variants that are strongly correlated with the first association, termed the exposure variable, to test for causal effects on an outcome. As genetic variants are fixed from conception, MR can also enable investigation of causality in the absence of environmental confounding and reverse causation.

**Figure 1:**
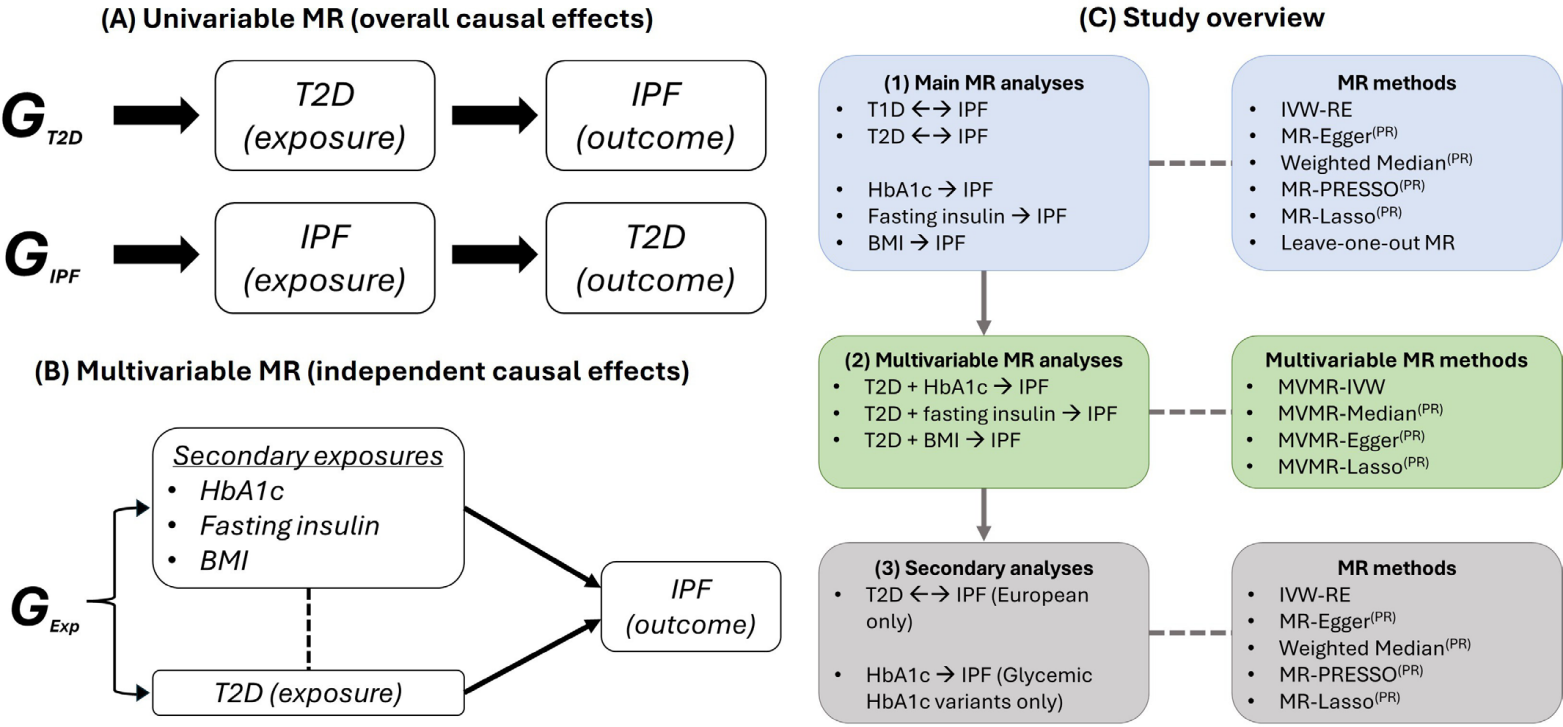
An overview of study design and analytical approaches. (A) Example directed acyclic graphs (DAGs) presenting a bidirectional Mendelian randomisation (MR) study design testing for causality between type 2 diabetes (T2D) and idiopathic pulmonary fibrosis (IPF). In each DAG, arrows indicate the directionality of the potential causal relationship that is being tested. Genetic variants that are strongly correlated with an exposure variable (G_T2D_ for a T2D exposure, G_IPF_ for an IPF exposure) are used as instrumental variables to test for causal effects on particular outcome (IPF and T2D, respectively). (B) A DAG presenting the multivariable MR design applied in this study. Genetic variants associated with multiple correlated exposures (G_Exp_) (in this example: T2D, glycated haemoglobin level (HbA1c), fasting insulin and body mass index (BMI)) are combined and integrated into the MR analysis framework to test for direct causal effects of each exposure on a single outcome (IPF). (C) A diagrammatic overview of this study, including potential causal relationships tested in main, multivariable and secondary analyses and MR methods used at each stage. IVW-RE, random-effects inverse-variance weighted; T1D, type 1 diabetes.

The main aim of this study was to estimate if T1D or T2D (exposures) are on the causal pathway for IPF (outcome) using a two-sample MR approach. MR analyses were also performed to investigate for any causal effects of IPF (exposure) on T1D or T2D (outcomes). Additional MR analyses were performed to assess for causal effects of continuous exposures representing DM-related factors, including average blood glucose (exposure variable: glycated haemoglobin level (HbA1c)), insulin signalling (exposure variable: fasting insulin level) and obesity (exposure variable: body mass index (BMI)) on IPF (outcome). Given the pathophysiological complexity of the T2D trait, multivariable MR analyses were also performed to assess for independent causal effects of T2D and these factors (HbA1c, fasting insulin and BMI) on IPF. A study overview is presented in figure 1.

## Methods

Robust causal estimation and control of confounding in MR analyses require genetic instruments that are

1. reliably associated with the investigated exposure (the *relevance* assumption);
2. independent of any confounders that influence both the exposure and the outcome (the *independence* assumption);
3. only associated with the outcome through the effects of the exposure (the *exclusion restriction* assumption).^11^

Pleiotropy is the occurrence of a single genotype that influences multiple distinct phenotypes. Horizontal pleiotropy can result in genetic instruments influencing the outcome through pathways outside of those acting through the exposure, which can confound MR analyses through violation of the exclusion restriction assumption.^11^ A range of MR methods have been developed that can provide pleiotropy-robust causal estimates.^12^ These methods often provide pleiotropy-robust causal estimates using different statistical approaches and each has its own limitations, therefore applying a variety of complementary methods is advantageous.^13^

A two-sample MR study design involves estimating associations between genetic variants and the exposure variable in one dataset, then estimating associations between the same genetic variants and an outcome in a different dataset.^12^ A two-sample approach therefore enables investigation of causal relationships between variables that have not been measured in the same cohort. Two-sample MR can also be applied in a multivariable setting, where instruments for multiple correlated exposures can be integrated to enable estimation of independent causal effects of each exposure on an outcome.^14^

### Study populations

Genetic association summary statistics were retrieved from genome-wide association studies (GWAS) of T1D^15^ (6683 European ancestry cases, 12 173 European ancestry controls, 2601 European ancestry affected sibling pair families and 69 European ancestry trios), T2D^16^ (61 714 European ancestry cases, 1178 Pakistani ancestry cases, 593 952 European ancestry controls and 2472 Pakistani ancestry controls), IPF risk^17^ (4125 European ancestry cases and 20 464 European ancestry controls), HbA1c^18^ (sample size: 88 355 of European ancestry), fasting insulin^19^ (sample size: 151 013 of European ancestry) and BMI^20^ (sample size: 681 275 of European ancestry). A secondary bidirectional analysis for T2D and IPF was performed using data from a smaller GWAS of only European ancestry participants^21^ (12 931 European ancestry cases and 57 196 European ancestry controls). Using GWAS with the same ancestry for both exposure and outcome aimed to provide causal estimates without potential confounding that can occur due to population stratification, which would violate the *independence* MR assumption.^22^ An additional secondary analysis of HbA1c on IPF used a subset of functionally glycaemic (associated with glycaemic traits as defined by Wheeler *et al*^18^) HbA1c variants. This secondary analysis aimed to check whether estimates calculated using the main HbA1c exposure were affected by genetic variants influencing HbA1c level through erythrocyte or haemoglobin biology.

In the GWAS used to select genetic instruments for IPF, all IPF cases were diagnosed using American Thoracic Society (ATS)/European Respiratory Society (ERS) guidelines.^17 23^ No participants in the HbA1c or fasting insulin GWAS cohorts had a diagnosis of T1D or T2D, reported use of treatments for DM, or had blood glucose measurements meeting DM diagnosis criteria (fasting glucose ≥ 7 mmol/L, 2hGlu ≥ 11.1 mmol/L or HbA1c ≥ 6.5%).^18 19^ Exclusion of participants with DM from HbA1c and fasting insulin GWAS cohorts enables genetic associations with these factors to be identified without complex effects resulting from DM-relevant treatments.

### Genetic instrument selection

Selected genetic variants were required to have strong associations (p<5×10^-8^) with exposure variables to avoid violation of the *relevance* MR assumption. A clumping approach was used to select independent signals (r^2^<0.001 within a 10 000 kb window) using the 1000 Genomes Project reference panel. All genetic variants were required to be sufficiently strong instruments (F>10) for each exposure to avoid weak instrument bias. Genetic variants selected to instrument an exposure that were missing from outcome datasets were replaced with suitable proxies (R^2^>0.7) where available.

### Statistical analyses

A random-effects inverse-variance weighted (IVW-RE) MR method^24^ was used to estimate the overall effect of each exposure on outcomes using the *MendelianRandomization* R package. MR-Egger^25^ and weighted median^26^ (MR-WM) MR methods were used here to provide pleiotropy-robust causal estimates in a sensitivity analysis. MR-Egger uses meta-regression to estimate causal effects adjusted for directional pleiotropy, whereas MR-WM allows for pleiotropic effects in up to 50% of instruments. MR-PRESSO^27^ and MR-Lasso^28^ are extensions of the IVW method that identify and remove likely pleiotropic genetic instruments prior to calculating causal estimates. MR-PRESSO identifies likely pleiotropic instruments through variant contribution to heterogeneity in the IVW meta-analysis of variant-specific MR estimates, whereas MR-Lasso identifies invalid instruments by estimating genetic effects on the outcome that bypass the exposure.^12 28^ Leave-one-out MR was additionally performed for all main analyses (figure 1C) to investigate if individual variants were independently driving or distorting overall causal estimates. All univariable MR methods, other than MR-PRESSO, have multivariable equivalents that were applied in MVMR (MVMR-IVW, MVMR-Median, MVMR-Egger and MVMR-Lasso). In MVMR, the same genetic variants used for each exposure in univariable analyses are combined into one multivariable genetic instrument (figure 1B).^14^ An additional variant clumping step was then applied to filter out any duplicate variants that are associated with multiple exposure variables.

## Results

### Selection of genetic instruments

In main MR analyses, 35 genetic variants were selected as instruments for T1D, and 118 variants as instruments for T2D. Genetic instrument sets were similarly identified for HbA1c level (37 variants), fasting insulin (36 variants) and BMI (131 variants). All selected variants for these exposures had corresponding GWAS summary results for IPF. For MR analyses with IPF exposure, many variants were missing from outcome datasets with no appropriate proxies (T1D outcome: 14/19 variants missing, T2D outcome: 9/19 variants missing). However, no IPF variants were missing from the European ancestry-only T2D GWAS.

### Estimation of causal effects

**Figure 2:**
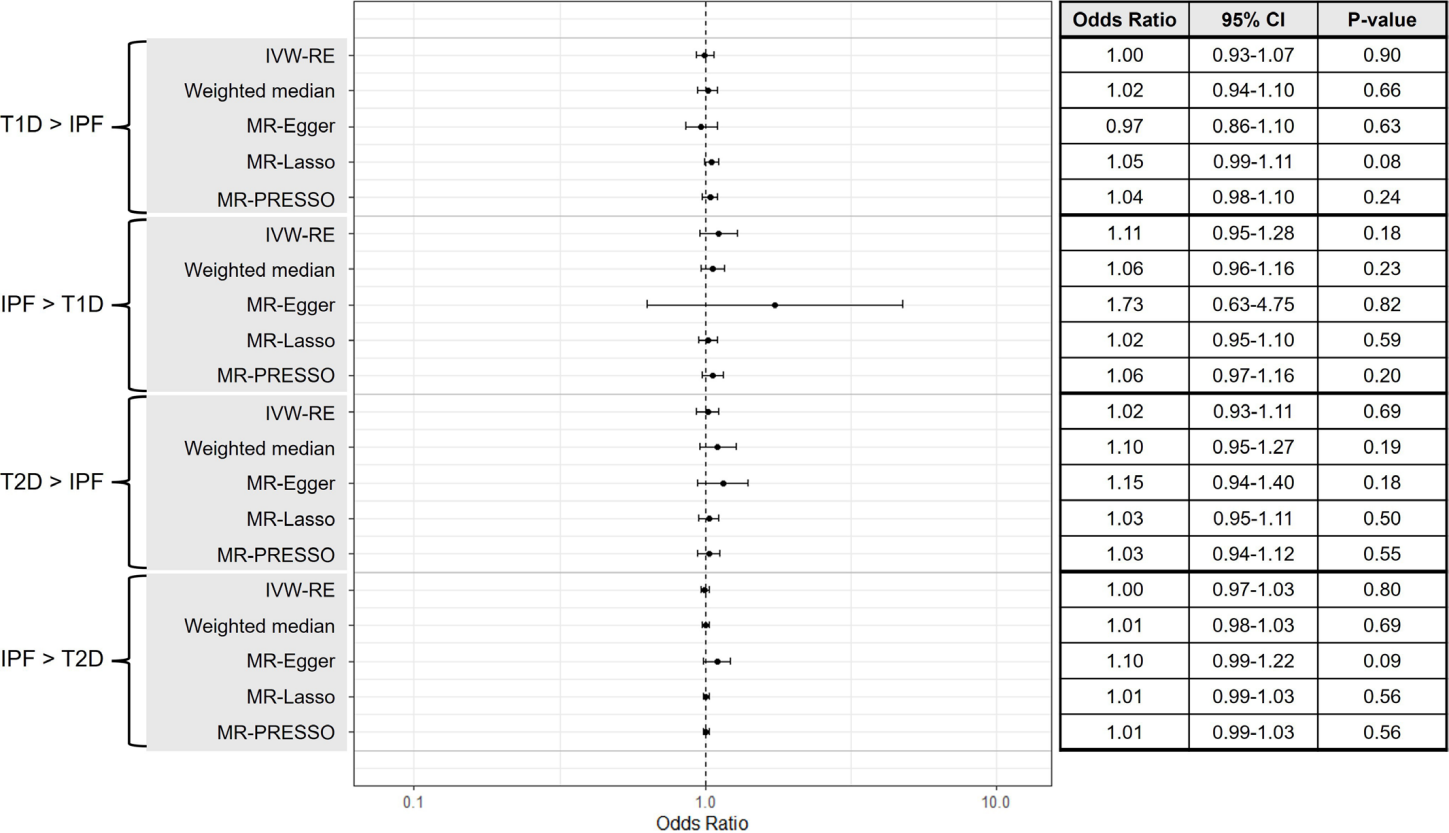
A forest plot and summary table of causal estimates from bidirectional Mendelian randomisation (MR) analyses testing for potential causal relationships between diabetes mellitus (DM) (type 1 diabetes (T1D) and type 2 diabetes (T2D)) and idiopathic pulmonary fibrosis (IPF). Error bars show 95% CI for overall estimates (OR) from each MR method (left). IVW-RE, random-effects inverse-variance weighted.

**Figure 3:**
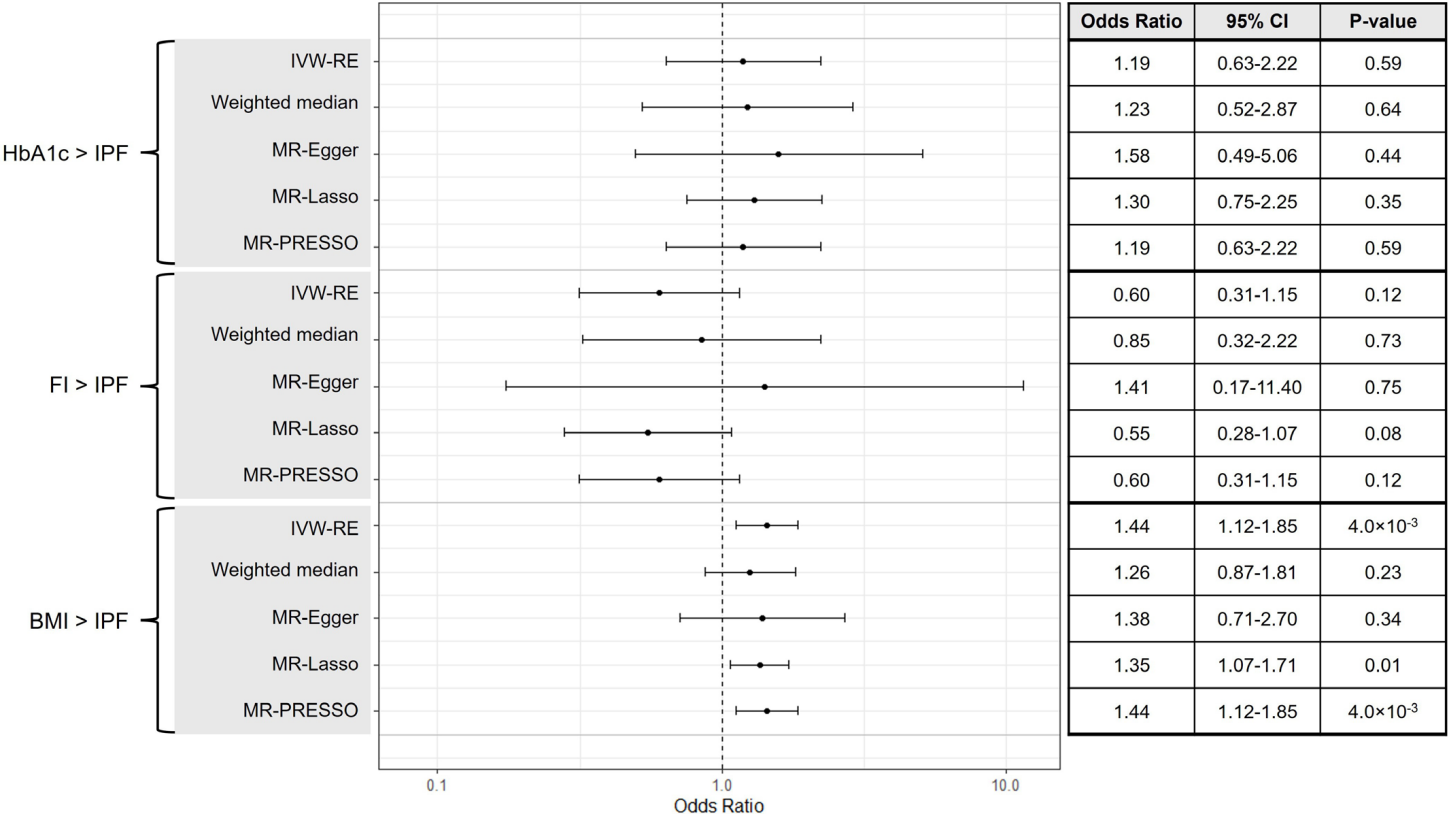
A forest plot and summary table of causal estimates from Mendelian randomisation (MR) analyses testing for potential causal effects of continuous diabetes mellitus (DM)-associated variables (glycated haemoglobin level (HbA1c), fasting insulin (FI) and body mass index (BMI)) on idiopathic pulmonary fibrosis (IPF). Error bars show 95% CI for overall estimates (OR) from each MR method (left). IVW-RE, random-effects inverse-variance weighted.

No causal effect was suggested of T1D on IPF (IVW-RE, OR=1.00, 95% CI 0.93 to 1.07, p=0.90) (figure 2). MR estimates similarly did not suggest causal effects of T2D on IPF (IVW-RE, OR=1.02, 95% CI 0.93 to 1.11, p=0.69). While there was heterogeneity present in analyses of T1D on IPF (Q=65.56, p=9.00×10^−4^, I^2^=48.10%), and, to a modest extent, of T2D on IPF (Q=148.43, p=0.03, I^2^=21.20%), results from pleiotropy-robust MR methods were consistent with those of IVW-RE (figure 2 and figure 3). Further, removal of likely pleiotropic variants completely removed the heterogeneity in analyses of T1D on IPF (MR-Lasso, Q=33.22, p=0.36, I^2^=6.7%), and T2D on IPF (MR-Lasso, Q=105.43, p=0.60, I^2^=0%), but MR estimates were consistent with those calculated using the IVW-RE approach. Leave one-out MR analyses of T1D on IPF (online supplemental figure 1), and T2D on IPF (online supplemental figure 2), did not identify any genetic variants that were likely to be independently driving or distorting overall causal estimates. Findings from secondary analyses of T2D on IPF with European ancestry-derived genetic instruments also aligned with main MR analyses (IVW-RE, OR=0.99, 95% CI 0.89 to 1.11, p=0.92) (online supplemental figure 3).

No causal effects of IPF on T1D (IVW-RE, OR=1.11, 95% CI 0.95 to 1.28, p=0.18) or T2D (IVW-RE, OR=1.00, 95% CI 0.97 to 1.03, p=0.80) were identified when the MR approach was applied in this direction (figure 2). Substantial was present in both the analysis of IPF on T1D (Q=21.45, p=3.00×10^−4^, I^2^=81.40%) and of IPF on T2D (Q=20.51, p=0.02, I^2^=56.10%). Removal of invalid instruments with MR-PRESSO and MR-Lasso completely removed heterogeneity in analyses of both T1D (MR-PRESSO, Q=2.82, p=0.42, I^2^=0.00%) and T2D (MR-PRESSO, Q=5.89, p=0.66, I^2^=0.00%) outcomes, but results were concordant with IVW-RE estimates. Findings from other pleiotropy-robust analyses were also consistent with IVW-RE results across both T1D and T2D outcomes. Leave-one-out MR analyses of IPF on T1D (online supplemental figure 4) and T2D (online supplemental figure 5) did not identify any individual variants that altered overall causal estimates. Results from secondary MR analyses of IPF on T2D using a European ancestry-derived genetic instrument were concordant with estimates from main analyses (IVW-RE, OR=0.97, 95% CI 0.93 to 1.01, p=0.18) (online supplemental figure 6).

### Estimation of causal effects using continuous exposures and MVMR

**Figure 4:**
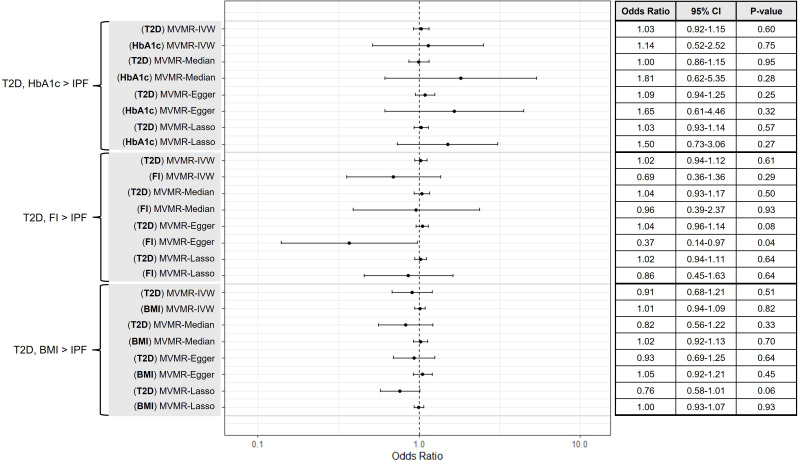
A forest plot and summary table of causal estimates from multivariable Mendelian randomisation (MVMR) analyses testing for direct causal effects of type 2 diabetes (T2D) and continuous diabetes mellitus (DM)-associated variables (glycated haemoglobin level (HbA1c), fasting insulin (FI), and body mass index (BMI)) on idiopathic pulmonary fibrosis (IPF). Error bars show 95% CI for overall estimates (OR) from each Mendelian randomisation method (left).

We found no evidence suggesting that HbA1c is on the causal pathway for IPF (IVW-RE, OR=1.19, 95% CI 0.63 to 2.22, p=0.59) (figure 3). No significant heterogeneity was detected in this analysis (Q=47.12, p=0.10, I^2^=23.60%). Leave-one out MR analysis also did not identify any individual variants that were independently driving or distorting overall causal estimates (online supplemental figure 6). Results of MVMR analyses did not suggest any independent effects of T2D (MV-IVW, OR=1.03, 95% CI 0.92 to 1.15, p=0.60) or HbA1c (MV-IVW, OR=1.14, 95% CI 0.52 to 2.52, p=0.75) on IPF (figure 4). Estimates calculated using pleiotropy-robust methods were consistent across univariate and MVMR analyses. Outcomes from secondary analyses of HbA1c on IPF using a subset of glycaemic HbA1c variants were consistent with findings from main HbA1c analyses (IVW-RE, OR=2.78, 95% CI 0.97 to 8.02, p=0.06) (online supplemental figure 3).

Results did not suggest that fasting insulin level is likely to be causally linked to IPF (IVW-RE, OR=0.60, 95% CI 0.31 to 1.15, p=0.12) (figure 3). No heterogeneity was found to be present (Q=35.72, p=0.43, I^2^=2.00%) and estimates from pleiotropy-robust analyses were consistent with those of IWV-RE. In addition, leave-one-out MR analyses did not highlight any individual variants that were independently driving or distorting causal estimates (online supplemental figure 7). Results of MVMR analyses did not generally indicate any independent effects of T2D (MVMR-IVW, OR=1.02, 95% CI 0.94 to 1.12, p=0.61) or fasting insulin (MVMR-IVW, OR=0.69, 95% CI 0.36 to 1.36, p=0.28) on IPF (figure 4). An independent protective effect of fasting insulin was indicated by MVMR-Egger results (OR=0.37, 95% CI 0.14 to 0.97, p=0.04); however, these results were not supported by MVMR-IVW, MVMR-Median or MVMR-Lasso analyses so are unlikely to represent a genuine causal relationship.

Estimates obtained by IVW-RE suggested a causal effect of BMI on IPF (IVW-RE, OR=1.44, 95% CI 1.12 to 1.85, p=4.00×10^−3^) (figure 3). No significant heterogeneity was present (Q=152.13, p=0.09, I^2^=14.50%) and leave-one-out MR did not identify any individual variants that were driving or distorting overall causal estimates (online supplemental figure 8). Results from the IVW-RE analysis were supported by MR-PRESSO (no invalid instruments detected) and MR-Lasso (OR=1.35, 95% CI 1.07 to 1.71, p=0.01). MR estimates from weighted median (OR=1.26, 95% CI 0.87 to 1.81, p=0.23) and MR-Egger methods (OR=1.38, 95% CI 0.71 to 2.70, p=0.34) were concordant with IVW-RE but were not statistically significant, which may be due to the lower statistical power of these methods. However, results of MVMR analyses did not suggest any independent effects of T2D (MVMR-IVW, OR=0.91, 95% CI 0.68 to 1.21, p=0.51) or BMI (MVMR-IVW, OR=1.01, 95% CI 0.94 to 1.09, p=0.82) on IPF (figure 4). Estimates obtained using pleiotropy-robust methods were consistent with those of MVMR-IVW.

## Discussion

The results of this study suggest that DM is unlikely to be in the causal pathway of IPF, either directly (T1D or T2D), or through related factors (HbA1c and fasting insulin). A positive effect was suggested of BMI on IPF, although this effect did not persist in multivariable MR (MVMR) analyses where BMI and T2D were modelled together.

This study did not identify any evidence to suggest that T1D is directly causally associated with IPF, which contradicts the findings of recent MR studies suggesting a risk-increasing effect of T1D on IPF.^29^ Our study uniquely identified genetic instruments for IPF using a GWAS cohort of IPF cases that were diagnosed following ATS/ERS guidelines.^17 23^ Compared with these previous studies, the GWAS cohort used to select IPF genetic instruments included a larger number of IPF cases (4125 compared with 1028). The lack of causal effects in either direction between T2D and IPF is consistent with the findings of previous studies.^29 30^ Our investigation provides a more comprehensive assessment of possible causal pathways by targeting specific pathways between T2D and IPF. Use of HbA1c instruments has been demonstrated to be superior to diabetes instruments in strength and variance explained^31^ and enables further insights into potentially interacting disease mechanisms. Inclusion of genetic instruments for HbA1c, fasting insulin and BMI as exposures alongside T2D therefore strengthens our study. The null effects of HbA1c on IPF do not align with previously proposed mechanisms of systemic hyperglycaemia promoting lung fibrosis through disrupted DNA repair and increased senescence.^8^ Results of univariable MR analyses suggested a positive effect of BMI on IPF; however, no independent effects of T2D or BMI on IPF were indicated by MVMR results. A previous study has suggested that patients with IPF are generally normally nourished or obese,^32^ but lower BMI is associated with poorer disease outcomes.^33^ Given the complexity of factors contributing to BMI, the pathway by which BMI could influence IPF is unclear.

This investigation applied a robust MR approach following current MR guidelines.^12^ As recommended, a variety of complementary pleiotropy-robust MR methods were selected to interrogate causal estimates for possible confounding.^13^ Use of binary exposures that are a dichotomisation of an underlying continuous variable, such as T2D diagnosis based on an HbA1c ≥ 6.5% threshold, can result in confounding.^34^ To account for this limitation, additional two-sample MR analyses were performed with continuous exposures (HbA1c, fasting insulin and BMI). Further, an MVMR approach was performed to distinguish the independent effects of correlated exposures (T2D, HbA1c, fasting insulin, and BMI). The inclusion of MVMR analyses of DM-associated factors therefore investigates potential causal pathways between DM and IPF more comprehensively than a standard MR approach.

This study has some limitations. While two-sample MR can provide valuable insights into disease aetiology, estimates are based on summarised data and generally can only represent overall effects. This approach cannot capture effects that may be specific to disease subtypes, disease stages, or complex effects due to gene–environment interactions and time-varying heritable risk factors. Use of disease status to define genetic instruments could also be considered an oversimplification of disease effects. Many variants were found to be missing from T1D and T2D outcome datasets in main analyses with an IPF exposure, which may have limited power to detect a true causal effect. Results also indicated the presence of significant heterogeneity in some analyses, which may reflect the biological complexity of some of the investigated traits. Overall, while MR is a powerful tool for assessing causal relationships, interpretation of these findings should consider other sources of evidence as recommended in current guidelines.^12^

The comorbid relationship between DM and IPF may alternatively result from shared factors affecting both traits, rather than directly causative effects. For example, T2D and IPF share risk factors such as smoking and ageing. It is therefore difficult to rule out potential confounding driven by shared upstream factors. Alternatively, factors such as shortened telomere length may independently drive both IPF and T2D. Shorter telomere length, which has been shown to be a causative factor in IPF,^35^ is associated with both T1D and T2D, insulin resistance and increased HbA1c level.^36^ As telomere length also decreases with increased age and cigarette smoking,^37 38^ this may also explain shared associations of these factors with T2D and IPF. Further, potential interaction of treatments across diseases may exist. Patients with IPF experiencing acute exacerbations are routinely treated with corticosteroids, which promote hyperglycaemia and diabetes.^39^ Similarly, metformin, which is the first-line oral therapy for the treatment of T2D, has been suggested to resolve lung fibrosis in cellular and animal models^40^ which may suggest the presence of shared or interacting disease pathways.

The findings of this study suggest that the comorbid relationship between DM and IPF is not driven by direct causative effects of either disease. Intrinsic mechanisms upstream of both traits or environmental effects may underlie this comorbid relationship. Shared mechanisms independently driving both traits may highlight opportunities for intervention and warrant further investigation.

## supplementary material

10.1136/thorax-2024-221472 online supplemental file 1

## Data Availability

Data are available upon reasonable request.
