# Supplementary material for: Assessing causal relationships between diabetes mellitus and idiopathic pulmonary fibrosis: a Mendelian randomisation study
Source: Thorax. 2024 Nov 29;80(3):e221472. doi: 10.1136/thorax-2024-221472 (PMC11877114; doi:10.1136/thorax-2024-221472)
Supplement: online supplemental file 1 [file thorax-80-3-s001.pdf]

Assessing causal relationships between Diabetes Mellitus and Idiopathic Pulmonary Fibrosis: A Mendelian Randomisation Study

Supplementary material

Supplementary Figure 1: A forest plot presenting causal estimates from leave-one-out MR analyses of T1D on IPF. The variant removed from each analysis is presented on the y-axis. Causal effect estimates are presented on the x-axis with beta (logOR) values, with error bars showing the 95% confidence intervals (95% CI) for each estimate.

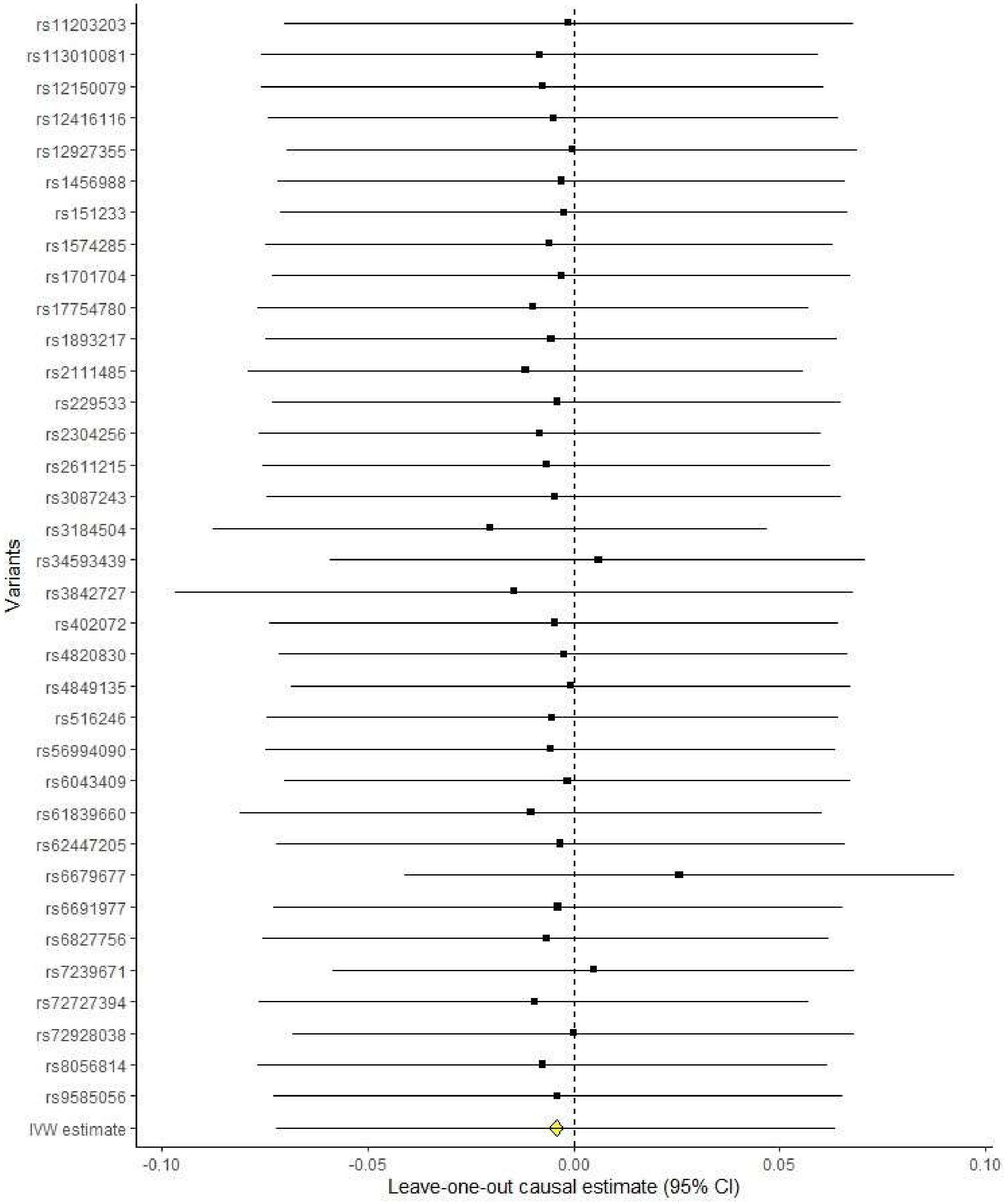

Supplementary Figure 2: A forest plot presenting causal estimates from leave-one-out MR analyses of T2D on IPF. The variant removed from each analysis is presented on the y-axis. Causal effect estimates are presented on the x-axis with beta (logOR) values, with error bars showing the 95% confidence intervals (95% CI) for each estimate.

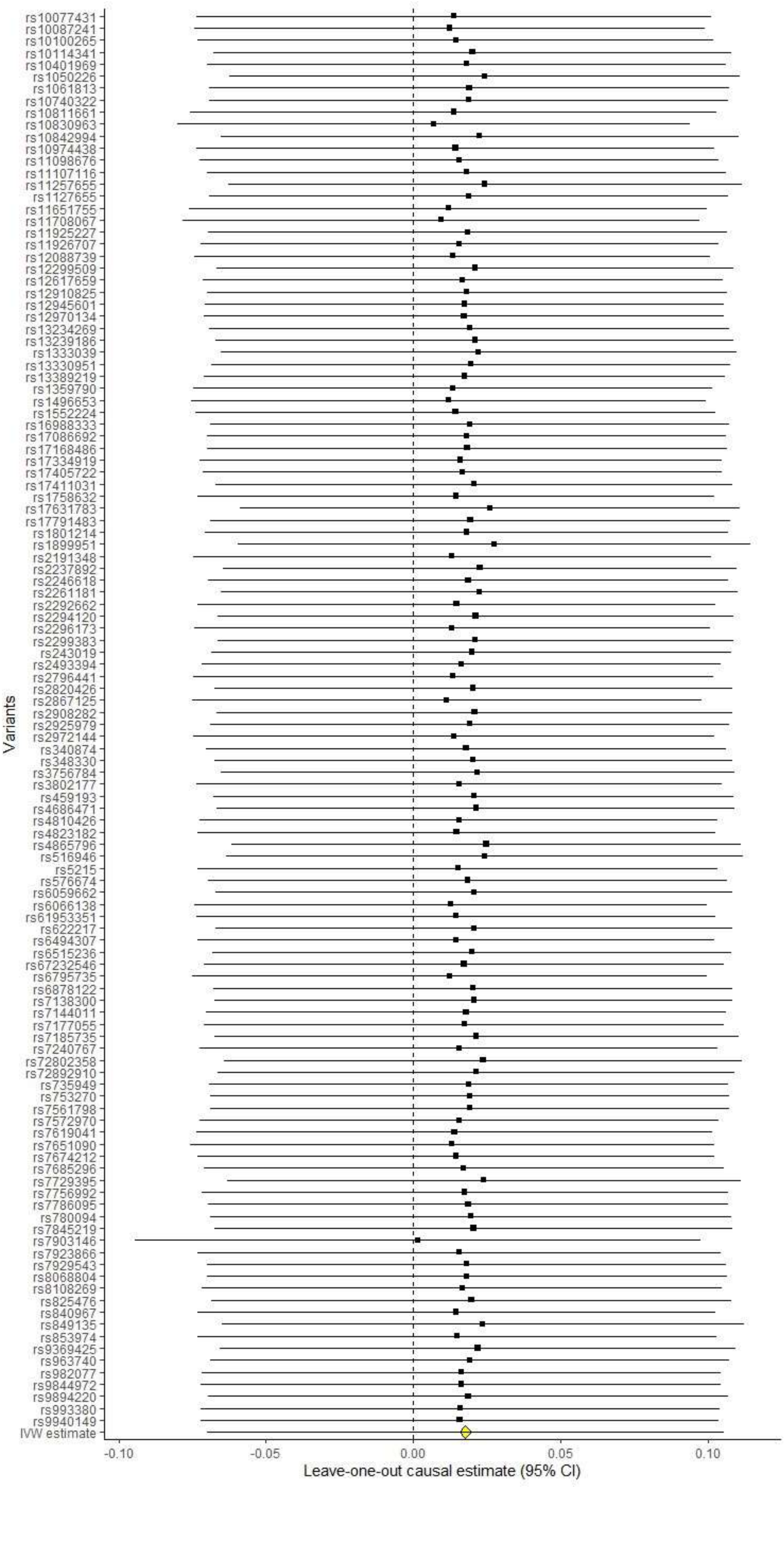

rs10077431
rs10087241
rs10100265
rs10114341
rs10401969
rs1050226
rs1061813
rs10740322
rs10811661
rs10830963
rs10842994
rs10974438
rs11098676
rs11107116
rs11257655
rs1127655
rs11651755
rs11708067
rs11925227
rs11926707
rs12088739
rs12299509
rs12617659
rs12910825
rs12945601
rs12970134
rs13234269
rs13239186
rs1333039
rs13330951
rs13389219
rs1359790
rs1496653
rs1552224
rs16988333
rs17086692
rs17168486
rs17334919
rs17405722
rs17411031
rs1758632
rs17631783
rs17791483
rs1801214
rs1899951
rs2191348
rs2237892
rs2246618
rs2261181
rs2292662
rs2294120
rs2296173
rs2299383
rs243019
rs2493394
rs2796441
rs2820426
rs2867125
rs2908282
rs2925979
rs2972144
rs340874
rs348330
rs3756784
rs3802177
rs459193
rs4686471
rs4810426
rs4823182
rs4865796
rs516946
rs5215
rs576674
rs6059662
rs6066138
rs61953351
rs622217
rs6494307
rs6515236
rs67232546
rs6795735
rs6878122
rs7138300
rs7144011
rs7177055
rs7185735
rs7240767
rs72802358
rs72892910
rs735949
rs753270
rs7561798
rs7572970
rs7619041
rs7651090
rs7674212
rs7685296
rs7729395
rs7756992
rs7786095
rs780094
rs7845219
rs7903146
rs7923866
rs7929543
rs8068804
rs8108269
rs825476
rs840967
rs849135
rs853974
rs9369425
rs963740
rs982077
rs9844972
rs9894220
rs993380
rs9940149
IVW estimate
Variants
-0.10
-0.05
0.00
0.05
0.10
Leave-one-out causal estimate (95% CI)

Supplementary Figure 3: A forest plot and summary table of causal estimates from MR analyses testing for potential causal relationships between T2D (European ancestry only) and IPF (bidirectional) and causal effects of HbA1c (glycemic variants only) on IPF. Error bars show 95% confidence intervals (95% CI) for overall estimates (Odds Ratio) from each MR method (left).

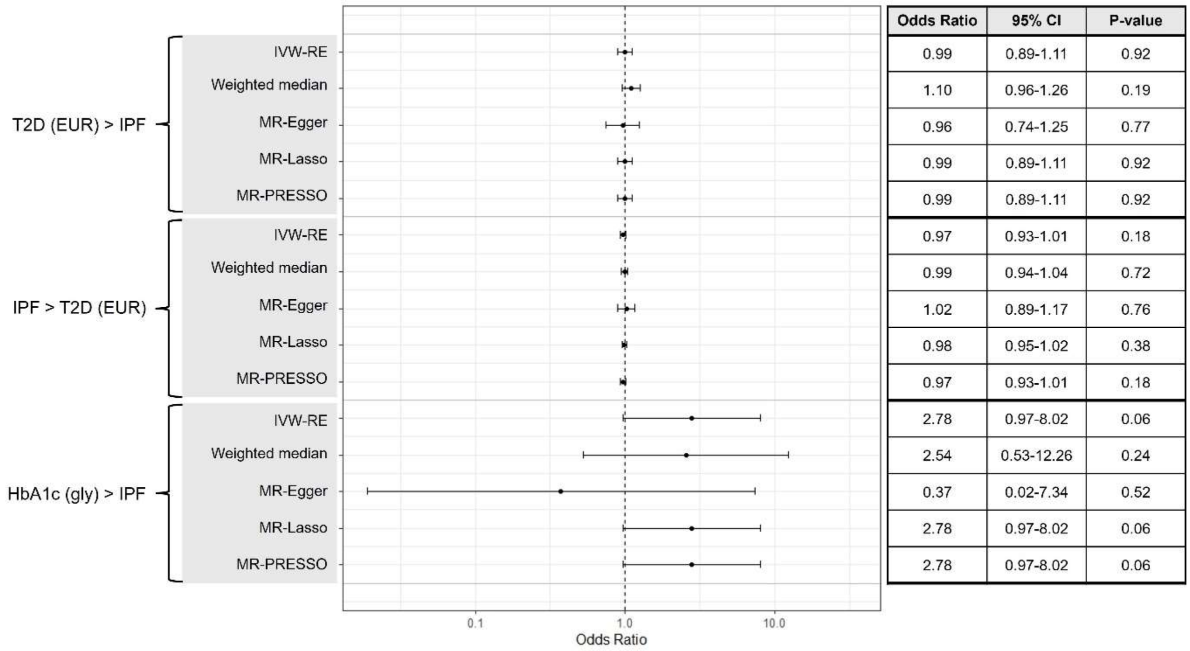

Odds Ratio	95% CI	P-value
0.99	0.89-1.11	0.92
1.10	0.96-1.26	0.19
0.96	0.74-1.25	0.77
0.99	0.89-1.11	0.92
0.99	0.89-1.11	0.92
0.97	0.93-1.01	0.18
0.99	0.94-1.04	0.72
1.02	0.89-1.17	0.76
0.98	0.95-1.02	0.38
0.97	0.93-1.01	0.18
2.78	0.97-8.02	0.06
2.54	0.53-12.26	0.24
0.37	0.02-7.34	0.52
2.78	0.97-8.02	0.06
2.78	0.97-8.02	0.06

Supplementary Figure 4: A forest plot presenting causal estimates from leave-one-out MR analyses of IPF on T1D. The variant removed from each analysis is presented on the y-axis. Causal effect estimates are presented on the x-axis with beta (logOR) values, with error bars showing the 95% confidence intervals (95% CI) for each estimate.

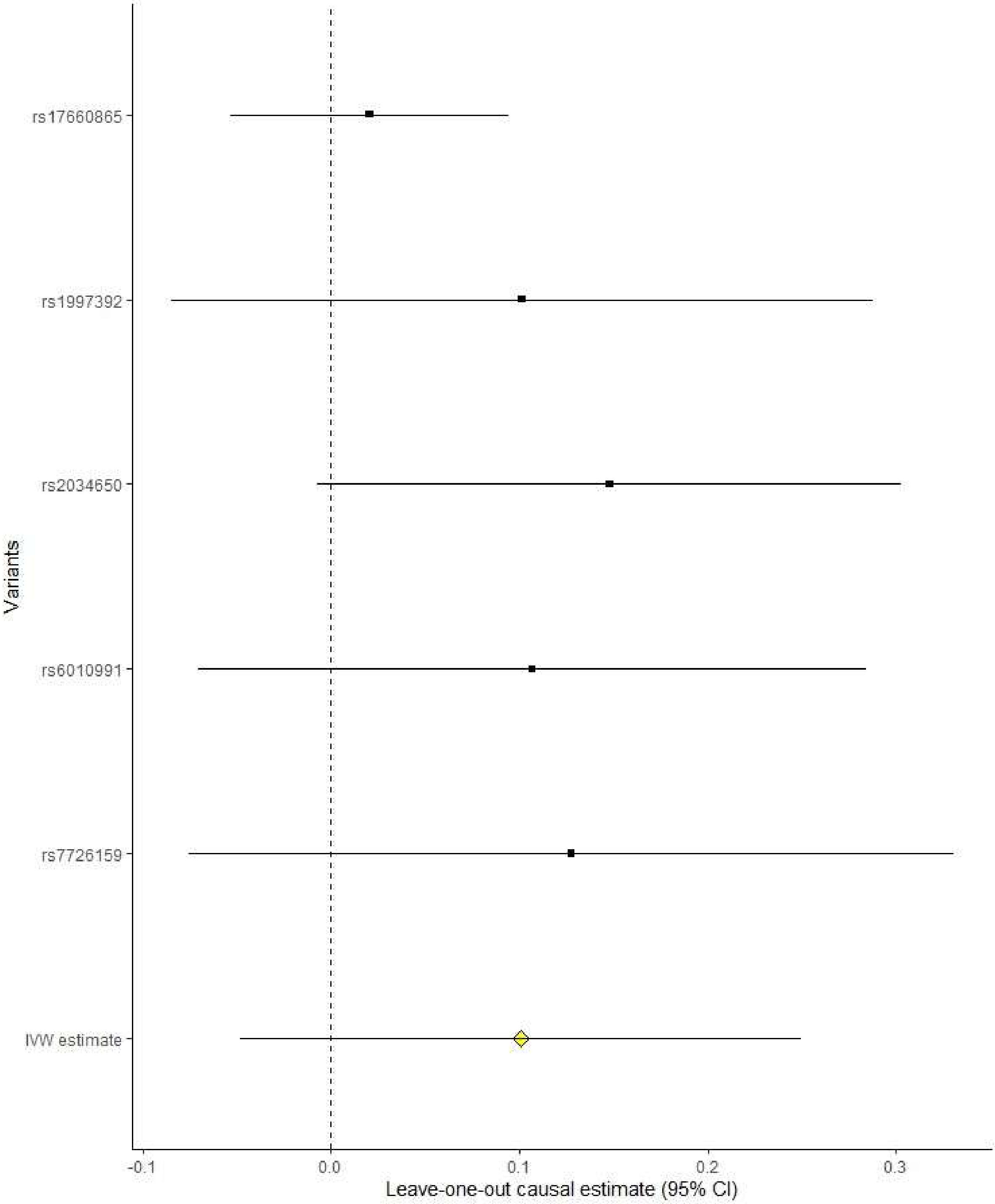

Supplementary Figure 5: A forest plot presenting causal estimates from leave-one-out MR analyses of IPF on T2D. The variant removed from each analysis is presented on the y-axis. Causal effect estimates are presented on the x-axis with beta (logOR) values, with error bars showing the 95% confidence intervals (95% CI) for each estimate.

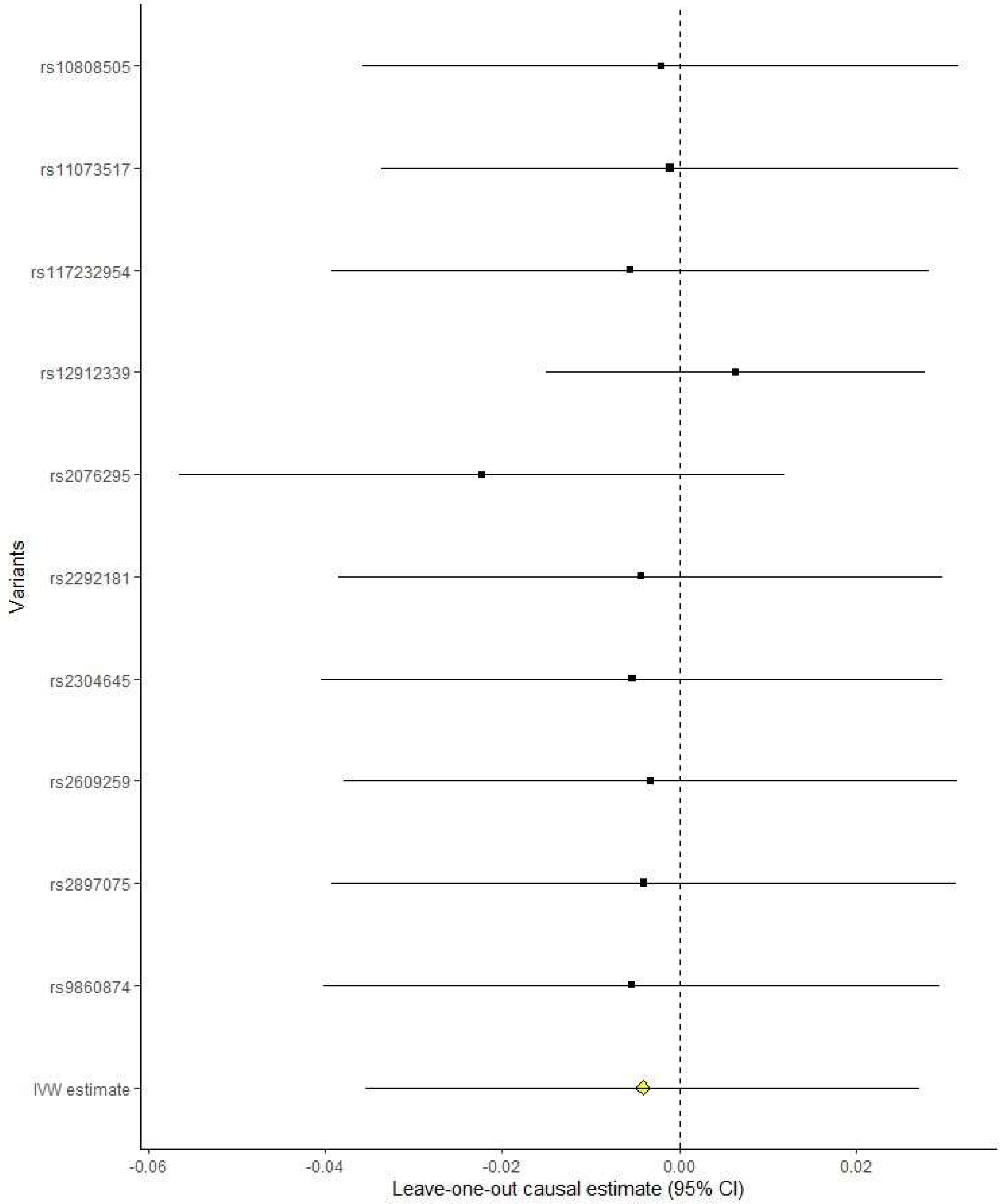

Supplementary Figure 6: A forest plot presenting causal estimates from leave-one-out MR analyses of HbA1c on IPF. The variant removed from each analysis is presented on the y-axis. Causal effect estimates are presented on the x-axis with beta (logOR) values, with error bars showing the 95% confidence intervals (95% CI) for each estimate.

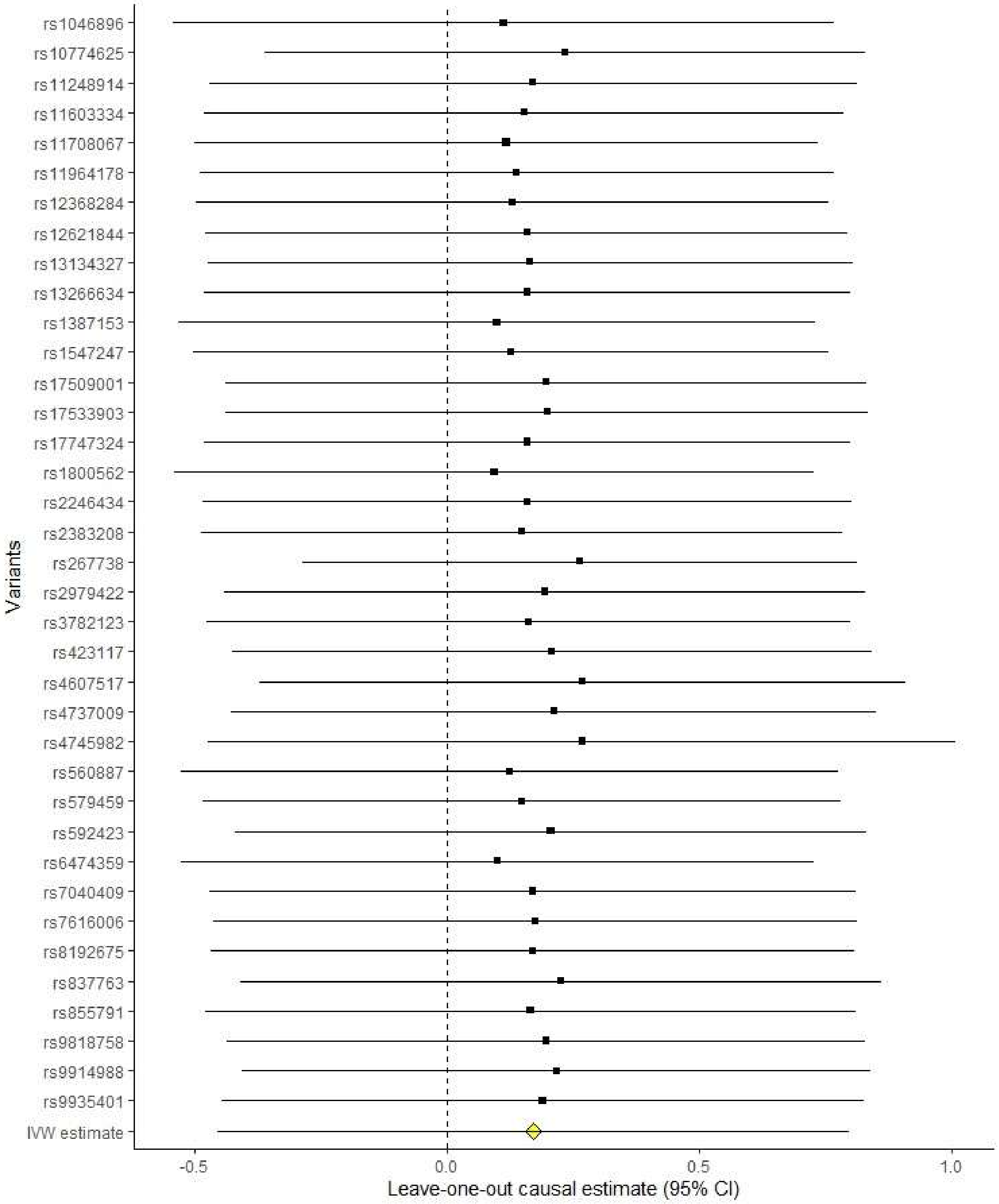

Supplementary Figure 7: A forest plot presenting causal estimates from leave-one-out MR analyses of fasting insulin on IPF. The variant removed from each analysis is presented on the y-axis. Causal effect estimates are presented on the x-axis with beta (logOR) values, with error bars showing the 95% confidence intervals (95% CI) for each estimate.

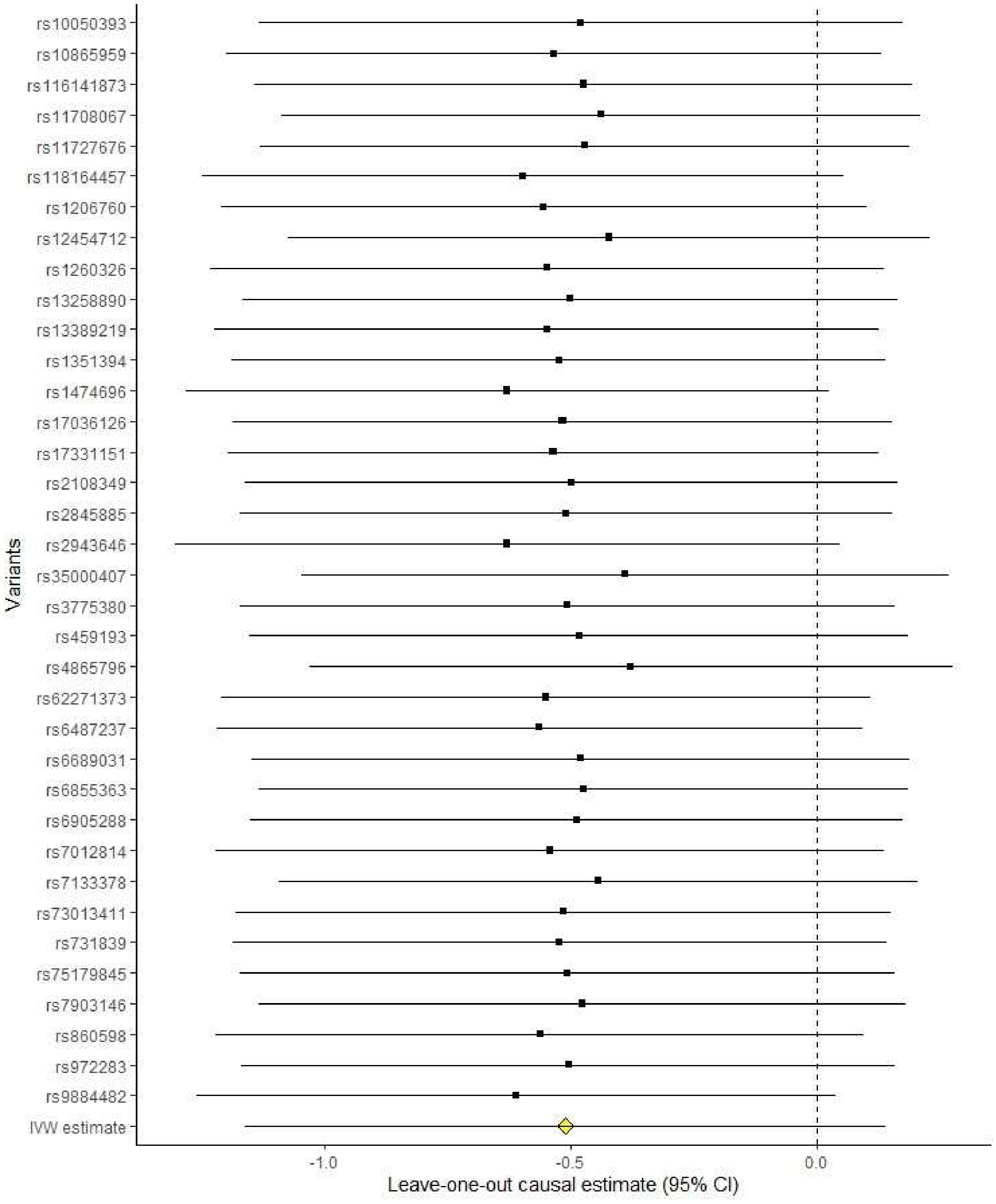

Supplementary Figure 8: A forest plot presenting causal estimates from leave-one-out MR analyses of BMI on IPF. The variant removed from each analysis is presented on the y-axis. Causal effect estimates are presented on the x-axis with beta (logOR) values, with error bars showing the 95% confidence intervals (95% CI) for each estimate.

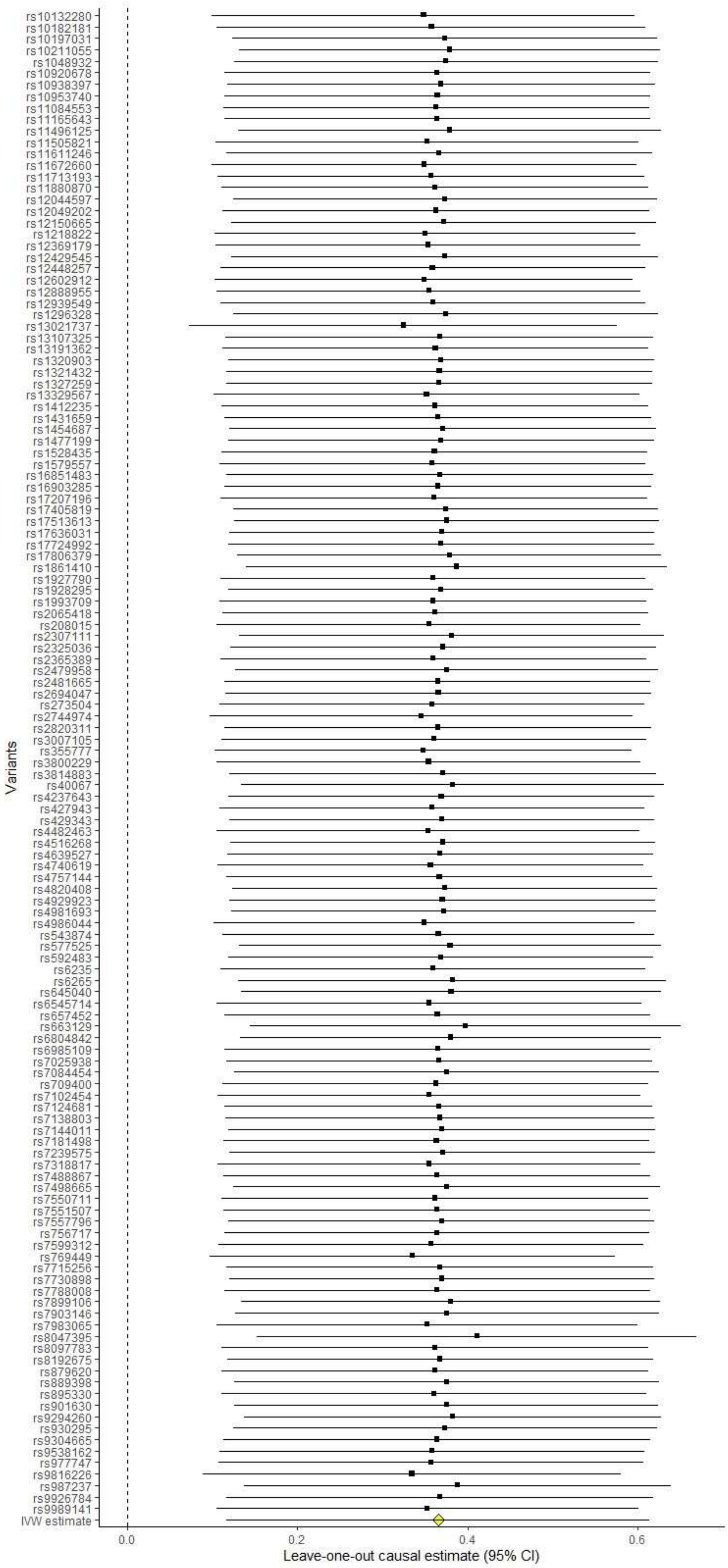

rs10132280
rs10182181
rs10197031
rs10211055
rs1048932
rs10920678
rs10938397
rs10953740
rs11084553
rs11165643
rs11496125
rs11505821
rs11611246
rs11672660
rs11713193
rs11880870
rs12044597
rs12049202
rs12150665
rs1218822
rs12369179
rs12429545
rs12448257
rs12602912
rs12888955
rs12939549
rs1296328
rs13021737
rs13107325
rs13191362
rs1320903
rs1321432
rs1327259
rs13329567
rs1412235
rs1431659
rs1454687
rs1477199
rs1528435
rs1579557
rs16851483
rs16903285
rs17207196
rs17405819
rs17513613
rs17636031
rs17724992
rs17806379
rs1861410
rs1927790
rs1928295
rs1993709
rs2065418
rs208015
rs2307111
rs2325036
rs2365389
rs2479958
rs2481665
rs2694047
rs273504
rs2744974
rs2820311
rs3007105
rs355777
rs3800229
rs3814883
rs40067
rs4237643
rs427943
rs429343
rs4482463
rs4516268
rs4639527
rs4740619
rs4757144
rs4820408
rs4929923
rs4981693
rs4986044
rs543874
rs577525
rs592483
rs6235
rs6265
rs645040
rs6545714
rs657452
rs663129
rs6804842
rs6985109
rs7025938
rs7084454
rs709400
rs7102454
rs7124681
rs7138803
rs7144011
rs7181498
rs7239575
rs7318817
rs7488867
rs7498665
rs7550711
rs7551507
rs7557796
rs756717
rs7599312
rs769449
rs7715256
rs7730898
rs7788008
rs7899106
rs7903146
rs7983065
rs8047395
rs8097783
rs8192675
rs879620
rs889398
rs895330
rs901630
rs9294260
rs930295
rs9304665
rs9538162
rs977747
rs9816226
rs987237
rs9926784
rs9989141
IVW estimate
Variants
0.0
0.2
0.4
0.6
Leave-one-out causal estimate (95% CI)
